# Editorial: Broadening our conceptual understanding of endogenous opioids in systems neuroscience

**DOI:** 10.3389/fnsys.2023.1212650

**Published:** 2023-06-13

**Authors:** Hugo A. Tejeda, Nicolas Massaly, Gregory Corder, Catherine M. Cahill

**Affiliations:** ^1^Unit on Neuromodulation and Synaptic Integration, National Institute of Mental Health, National Institutes of Health, Bethesda, MD, United States; ^2^Department of Anesthesiology and Perioperative Medicine, University of California, Los Angeles, Los Angeles, CA, United States; ^3^Department of Psychiatry, University of Pennsylvania, Philadelphia, PA, United States; ^4^Department of Psychiatry and Biobehavioral Science, University of California, Los Angeles, Los Angeles, CA, United States

**Keywords:** opioid peptides, opioid receptors, neuronal circuits, pain, reward, alcohol and substance use disorders, neuromodulation, respiratory depression

Endogenous opioid peptides and their receptors are critical mediators of various physiological and psychological processes, including motivation, affect, pain processing, cognition, stress-responsivity, and autonomic function. These opioid systems are embedded in neuronal circuits that subserve specific aspects of such processes and play a pivotal role in finely tuning behavioral outcomes. This is of relevance as dysfunction in endogenous opioid systems has been implicated in a plethora of neuropsychiatric disorders characterized by alterations in several of symptom clusters. To date, opioid receptor systems remain a promising therapeutic target for a variety of neuropsychiatric disorders and are an area of on-going development.

As a field, we have achieved significant advancements in our understanding of the cellular and molecular underpinnings of endogenous opioids in regulating cellular activity and behavior. However, there remain critical knowledge gaps in our understanding of how endogenous opioid systems are embedded in neuronal circuits and how these systems regulate emergent properties of brain function. Recent advances in technology have permitted a thorough dissection of endogenous opioid systems with cell type, pathway, and subcellular resolution. Accordingly, progress has provided us with a better understanding of endogenous opioid system engagement in regulating neuronal systems that subserve the aforementioned physiological and psychological processes. Despite those critical breakthroughs, further efforts are necessary to unravel the precise granularity with which endogenous opioid systems dysregulation alters pathways necessary for the development and maintenance of pathophysiological states in mental health disorders.

With the present collection of articles in this Research Topic, we provide recent advancements in our understanding of endogenous opioids from receptor signaling to neuronal circuits and highlight future avenues and opportunities for research aiming at elucidating novel targets and approaches to treat neurological and psychiatric disorders.

Gamble et al. highlight recent advancements in uncoupling cellular mechanisms mediating beneficial pharmacological effects such as treatment of pain and receptor signaling that leads to various unwanted effects such as analgesic tolerance, physical dependence and activation of reward circuitry. They discuss the relatively unappreciated interaction of mu opioid receptors (MOR) with receptor tyrosine kinases (RTKs), including RTKs transactivation of MOR, and the potential therapeutic implication for targeting RTK to enhance the safety and analgesic profile of MOR agonists. There is a strong case to be made that RTK inhibitors may be a co-treatment therapy that will allow opioid mediated analgesic properties with attenuated negative outcomes associated with chronic opioid pharmacotherapies.

Adhikary and Williams provide a synthesis of the recent advancement on cellular tolerance induced by chronic opioid receptor activation. In addition, they present insights into how tolerance can be manifested at the neuronal systems level. One highlight in their article is that tolerance is not mediated by a single regulatory mechanism but rather by various adaptations in cellular processes and circuits. In their review they discuss pre- and post-synaptic mechanisms underlying cellular tolerance to chronic morphine in multiple brain regions, and how the pharmacological properties of opioids, including their potency and efficacy, produce distinct adaptations that lead to tolerance.

The ventrolateral periaqueductal gray area (PAG) is well recognized for its importance in descending modulation of pain (pre-clinical; Lubejko et al.), or conditioned pain modulation (clinical). McPherson and Ingram review the importance of the opioid systems in descending pain modulation and how opioids contribute to the cellular and circuit diversity within the PAG implicated in pain transmission and opioid analgesia. The granularity on the description of circuit afferent, cell type specificity, and cellular signaling provides a cohesive summary of our current knowledge on this specific area of research. One of the important messages for future direction in this area is the potential for optimizing novel target drug development by taking advance of our knowledge of cellular heterogeneity in the PAG and other regions implicated in descending pain modulation.

The manuscript by Lubejko et al. further addresses the importance of opioid systems in analgesia and pain treatment. In this article, they emphasize the potential of neurostimulation as a novel treatment strategy that mitigates side effect profiles associated with small molecule MOR ligands. Various neurostimulation techniques have been proposed to treat pain including deep brain stimulation (DBS), spinal cord stimulators, vagal nerve stimulation and transcranial direct current stimulation (tDCS). This paper highlights evidence supporting the effectiveness of various neurostimulation techniques and how they may engage opioid systems to produce their beneficial effect in pain management. The authors also provide a comprehensive overview of the various brain regions and circuits where pain transmission is processed and modulated by opioids, as well as a future outlook of the next generation of safe, effective, and technologically-innovative clinical treatments.

Limoges et al. provide us with a detailed description of the architecture and function of the dynorphin kappa-opioid receptor system, specifically in amygdala circuits. This review presents evidence from current literature demonstrating that the dynorphin kappa-opioid receptor system plays a pivotal role in controlling many aspects of behavior, including aversive learning, pain-related, and alcohol and drug-seeking behaviors. Embedded in this article are comprehensive illustrations of the various neural circuit inputs onto dynorphin kappa opioid receptor expressing cells within the basolateral and central amygdala as well as targets of these neurons, providing a framework for how the kappa opioid system may contribute to neuropsychiatric disorders.

Adding to this article, Reeves et al. provide a comprehensive overview of the role of opioid systems in regulating synaptic transmission and intrinsic excitability across the nervous system. In their review they summarize the current literature to present a detailed description of how endogenous opioids finely tune information flow in discrete circuits, providing a cellular basis wherein opioid-mediated transmission may shape circuits at the systems level.

There is a pressing need to understand how opioids produce reinforcement and drug seeking behaviors, especially in the context of pain treatment, as chronic pain has been identified as a risk factor for developing an opioid use disorder. Higginbotham et al. provide a review on how pain or prolonged opioid exposure modifies reward circuitry and changes in opioid receptor function. There is strong evidence that persistent pain negatively impacts reward sensitivity and mood. Together, pain modulation of reward sensitivity and mood contributes to susceptibility in initial opioid misuse and the development of opioid use disorder. They provide a strong message that the ability to curb the opioid crisis will require more understanding of how pain and opioid-induced adaptations alter functional neurocircuitry. Indeed, much of this research is still in its infancy. Complementing this review is another by Rysztak and Jutkiewicz that comprehensively describe the mechanisms by which enkephalin peptides and enkephalin-expressing neuronal circuits mediate reward function, focusing on the modulation of mesolimbic dopamine circuitry. The authors further discuss alterations in those enkephalinergic systems in models of opioid use disorder.

Lastly, Maletz et al. share a compelling study in which they identify non-overlapping neuronal populations, expressing or lacking MOR, activated by morphine and hypoxia/hypercapnia in the Nucleus Tractus Solitarius. This original data report provides a deeper understanding of the circuits and neuronal populations involved in opioid-induced respiratory depression and may lead to further advancement for prevention and recovery of such events.

Overall, this compilation of papers recognized various future directions that need to be further explored. While there has been significant advancement in our understanding of cellular and synaptic level mechanisms that mediate opioid-regulated behaviors, there is much to be gained by furthering our understanding of how opioids regulate the activity of defined synapses and excitability to shape the activity of large-scale networks and emergent properties of the brain. Emerging research has shown that opioid receptor signaling is diverse and much more complex than what was previously appreciated (e.g. ligand-directed signaling or functional selectivity). However, how nuanced signaling by endogenous opioid systems regulate information flow in neuronal circuits is in large part unresolved. Further, future research is needed to develop a model that integrates opioid systems with other signaling modalities (e.g., RTKs, etc). This provides a basis for diversity of gene expression across cell types of the brain to influence not only where opioids may act, but also their function effect on circuits. Additionally, while the rapid rise in technology development has facilitated swift progress in the neuroscience field, further tools are needed to precisely dissect the functional anatomy and activity of the opioid systems. Those will be critical to delineate the effects of endogenous opioids on circuits with high spatiotemporal resolution helping us to better understand and solve essential questions. For instance, revealing activity dynamics of not only neurons expressing opioid peptides, but subsequent opioid peptide release and receptor-mediated signaling in models of affective pain or analgesia tolerance will help elucidating potential steps where endogenous opioid system dysfunction occurs. Together, this will provide critical insight necessary to develop novel opioid therapeutics for safer treatment for both the sensory and emotional experiences associated with pain. Another exciting trajectory of the field will be to understand the role of opioids in the context of other treatment modalities such as neuromodulation (e.g., DBS, tDCS) or rapid-acting antidepressants, which may in part exert their function via endogenous opioids or can synergize with opioid-based treatments. In conclusion, here we present a collection of articles focused on opioid systems in the context of systems neuroscience, which highlights developments and provide insights to the future of opioid research in this setting.

## Author contributions

All authors listed have made a substantial, direct, and intellectual contribution to the work and approved it for publication.

